# *Drosophila melanogaster*: a simple system for understanding complexity

**DOI:** 10.1242/dmm.041871

**Published:** 2019-10-01

**Authors:** Stephanie E. Mohr, Norbert Perrimon

**Affiliations:** 1 Department of Genetics, Harvard Medical School, Boston, MA 02115, USA; 2 Drosophila RNAi Screening Center, Harvard Medical School, Boston, MA 02115, USA; 3 Howard Hughes Medical Institute, Boston, MA 02115, USA

## Abstract

Understanding human gene function is fundamental to understanding and treating diseases. Research using the model organism *Drosophila melanogaster* benefits from a wealth of molecular genetic resources and information useful for efficient *in* *vivo* experimentation. Moreover, *Drosophila* offers a balance as a relatively simple organism that nonetheless exhibits complex multicellular activities. Recent examples demonstrate the power and continued promise of *Drosophila* research to further our understanding of conserved gene functions.

## Abstract (precis)

**Summary:** This Editorial outlines how research in *Drosophila* has and continues to help answer complex research questions in genetics, highlighting the value of this relatively simple model organism.

## Introduction

Following the completion of the Human Genome Project and the advances in sequencing technologies and bioinformatics, thousands of disease genes have been identified, paving the road to a revolution in medicine. Nevertheless, we face significant impediment to further progress: in the majority of cases, we do not understand how variants in a gene cause disease because the function of the gene itself is not well understood. Much of the knowledge we do have has come from fundamental studies and reflects an understanding of function at the cellular level. To learn more about human health and treat diseases, we must study complex biological activities in multicellular contexts. *Drosophila* provides an exemplary system in which to study gene functions in specific tissues and developmental stages, and under normal or perturbed conditions (Ugur et al., 2016). Here, we highlight recent examples that demonstrate how a convergence of technology and investigation can provide new insights into the mysteries of multicellular life. These examples show how years of tool development and experimentation in the fly are enabling unique and deep biological discoveries with translational implications. As *Drosophila* biologist Curt Stern noted in 1954: “Progress often proceeds best on the basis of past accomplishments. New questions may be asked on the basis of old experiments and sometimes answers are possible because of information already available” (Stern, 1954). Building on more than a century of accumulated knowledge – and taking advantage of established and emerging molecular genetic technologies – fly research is well positioned to remain a leading contributor to our understanding of how genes control complex biological activities.

## Mitochondria quality control

Studies of the *Drosophila* female germline have led to many fundamental discoveries in stem cell biology, egg formation, interaction between germline and soma, and cues deposited maternally to control embryonic patterning. A study by Lieber et al. (2019) documented a fascinating mechanism in the female germline that prevents the accumulation of deleterious mitochondrial mutations. Mitochondria have a high mutation rate and low levels of recombination of mitochondrial DNA (mtDNA). In both mammals and flies, a selection mechanism in the female germline prevents the accumulation of deleterious mutations (reviewed in Palozzi et al., 2018). Using wild-type mtDNA from *Drosophila* *yakuba* and mutant mtDNA from *Drosophila* *melanogaster* (Ma et al., 2014), Lieber et al. were able to visualize mtDNA selection in the *Drosophila* female germline using species-specific fluorescent *in situ* hybridization probes to distinguish wild-type and mutant mtDNA. Strikingly, the first step in this selection process is fragmentation of the mitochondria, leading to physical separation of mitochondrial genomes into smaller mitochondrial compartments. Fragmented mitochondria that contain mutant genomes are eliminated by mitophagy, resulting in an overall increase in wild-type mtDNA (Lieber et al., 2019). Further studies on the regulation of this fundamental mechanism are likely to provide important insights in diseases associated with mitochondrial dysfunction.

## Sugar and water regulation by hunger and thirst

Understanding how neurons are wired and how signals move through neuronal circuits to control behavior are important areas of neurobiology to which *Drosophila* is making fundamental contributions. Key to the progress made in recent years are the thousands of *Gal4* and split *Gal4* (Brand and Perrimon, 1993; Dionne et al., 2018) lines available in which genes or other DNA-encoded reagents can be expressed in a manner that is genetically defined and consistent across individuals. For neuroscience research, these resources allow single neurons or groups of neurons to be activated using *UAS-TrpA1* (Hamada et al., 2008) and allow for Ca^2+^ imaging using G-CaMP (a high-affinity Ca^2+^ probe containing a single GFP molecule) (Nakai et al., 2001). These tools have enabled the mapping of neuronal circuits that regulate specific behaviors, as exemplified by a study from Jourjine et al. (2016). Thirst and hunger are induced in response to a bodily state of dehydration or starvation, respectively, in order to regain homeostasis. How the internal state of an organism induces these behaviors has remained an open question. Jourjine et al. (2016) identified a *Gal4* driver expressed in the nervous system that, when combined with *UAS-TrpA1* to activate neurons, increased feeding. They then further refined the location of the relevant neurons using an intersectional approach in which they limited *Gal4* activity using the Gal4 repressor Gal80 (Suster et al., 2004). Subsequently, the group used a complementary RNA interference (RNAi)-based approach to identify genes relevant to thirst and found that hunger and thirst converge on the same set of neurons, suggesting that these neurons integrate the information and ‘weigh competing needs’ of the fly (Jourjine et al., 2016).

## Organ cross-talk and sex differences in physiology

Understanding sex differences in metabolism and physiology has become an area of increased interest. Studies in this area are relevant to human health, including potential differences between male and female disease susceptibility. Studies in *Drosophila* have led to a deep understanding of how sex chromosome number is interpreted to activate downstream pathways controlling morphological and behavioral differences between male and female flies. A recent study by Hudry et al. (2019) comparing gene expression in the guts of males and females revealed male-biased expression of enzymes involved in carbohydrate transport and utilization. Hudry et al. demonstrate that, surprisingly, this sex difference in gut gene expression is controlled by the adjacent male gonad, which produces the ligand Upd1. Upd1 activates JAK-STAT signaling in the enterocytes located in the adjacent intestinal subregion, upregulating the expression of sugar-metabolism-related genes and leading to cytosolic citrate production (Hudry et al., 2019). In this context, the role of citrate is twofold: citrate export promotes food consumption and is also transferred to adjacent testes to promote male gamete maturation. This striking inter-organ communication between testis and gut may have relevance for a spectrum of conditions in humans resulting from the abnormal arrangement of internal organs.

## Inter-organ growth coordination

Another important question for multicellular organisms is how the relative proportions of organs, limbs and so on are coordinated during development, even when one of the structures is damaged. A study by Boulan et al. (2019) took advantage of the *Drosophila* wing imaginal disc, a well-studied epithelial tissue composed of cells that are ‘set aside’ early in development and destined to form the wing during metamorphosis. Disruption of a ribosomal gene in the wing disc had a non-autonomous effect on growth of the eye imaginal disc. The group found that this process is controlled by Dilp8 (Garelli et al., 2012; Colombani et al., 2012), which is secreted when growth is inhibited and affects growth in other tissues via inhibition of the insect hormone ecdysone. Boulan et al. were able to further identify two relevant upstream factors: the bZIP-type transcription factor Xrp1, which is required for Dilp8 expression in slow-growing tissues, and the ribosomal protein RpS12, which acts as a sensor of tissue growth (Boulan et al., 2019). The study contributes to an increasingly clear picture of how tissue growth is coordinated both autonomously and non-autonomously, under normal and perturbed conditions.

## Modeling cancer

In many cases, modeling a disease can be accomplished through perturbation of single genes. In other cases, however, the most appropriate model would include multiple genetic perturbations. This seems particularly relevant for modeling cancer, as tumors are well documented to have multiple genetic changes. Bangi et al. (2019) reported the identification of a therapeutic strategy using a platform designed to model complex genetic changes identified by sequencing of an individual patient's tumor. Specifically, they used a combination of RNAi and ectopic expression to perturb nine different genes in a manner that parallels that identified in the tumor. The model was used to identify a treatment strategy that was then implemented clinically. The patient experienced a ‘progression-free’ period of 3 months and a partial response lasting 8 months. The treatment was not curative and the necessarily *n*=1 nature of such a personalized approach makes it difficult to draw general conclusions. Nevertheless, the fact that it was possible to develop and screen a *Drosophila* model that included perturbation of nine genes is impressive and offers some degree of hope for patients who suffer from complex cancers with poor prognosis.

## Concluding remarks

The extensive information we have about *Drosophila* provides a strong foundation on which to build a more complete mechanistic understanding of complex activities and answer as-yet-unanswered questions, including those that can only be addressed in whole-animal systems. With this expanding knowledge, we can seek out new means for preventing, controlling and treating human diseases. Although here we focused on examples related to uncovering gene function, *Drosophila* studies are also contributing to additional disease-relevant areas, including diagnosis of genetic diseases (reviewed by Bellen et al., 2019) and drug discovery or repurposing (e.g. see Ali et al., 2018). Technologies such as CRISPR knockout screens in human cells, single-cell RNA sequencing, and culture of tissue-specific organoids have expanded the range of questions that can be directly addressed using human cells. However, the availability of these technologies does not supplant the usefulness of model organisms; genetic models such as *Drosophila* remain relevant. In the area of single-cell sequencing, for example, the *Drosophila* system can provide comparison datasets that add confidence in the identification of new cell types and states, their markers, differentiation factors, and so on, similar to the way that fly functional genomic studies provide important comparative data for analyses of human genetic datasets. It would be unproductive to get caught up in debating whether this or that model system or approach is the best. Efficient study of conserved gene functions requires the use of both simple models such as *Drosophila* and more complex human-cell-based models, just as it requires the application of more than one experimental approach. Given the strength of *Drosophila* as a research system, there can be no doubt that the path ahead will continue to be informative and exciting.
